# Association of service facilities and amenities with adolescent birth rates in Mexican cities

**DOI:** 10.1186/s12889-023-16251-0

**Published:** 2023-07-10

**Authors:** Ariela Braverman-Bronstein, Dèsirée Vidaña-Pérez, Ana V. Diez Roux, Carolina Pérez Ferrer, Brisa N. Sánchez, Tonatiuh Barrientos-Gutiérrez

**Affiliations:** 1 Department of Epidemiology and Biostatistics, Dornsife School of Public Health, Drexel University, Philadelphia, PA USA; 2 Center for Survey Research and Evaluation, National Institute of Public Health, Cuernavaca, Mexico; 3 CONACYT, National Institute of Public Health, Cuernavaca, Mexico; 4 Center for Population Health Research, National Institute of Public Health, Av. Universidad 655, 62100 Cuernavaca, Mexico

**Keywords:** Adolescent pregnancy, Urban environment, Structural availability, Mexico

## Abstract

**Background:**

The association of the built environment and the structural availability of services/amenities with adolescent birth rates (ABR) has been overlooked in Latin America. We investigated the association of the availability, and changes in the availability, of services/amenities with ABR in 92 Mexican cities.

**Methods:**

We estimated ABR using data on live birth registration linked to municipality of residence at the time of birth from 2008–2017. The number of services/amenities were obtained from the National Statistical Directory of Economic Units in 2010, 2015, and 2020 and grouped as follows: education, health care, pharmacies, recreation, and on- and off-premises alcohol outlets. Data were linearly interpolated to obtain yearly estimates. We estimated densities per square km by municipality. We fitted negative binomial hybrid models, including a random intercept for municipality and city, and adjusted for other social environment variables.

**Results:**

After adjustment a 1-unit increase in the density of recreation facilities, pharmacies, and off-premises alcohol outlets within municipalities was associated with a 5%, 4% and 12% decrease in ABR, respectively. Municipalities with higher density of education, recreational and health care facilities had a lower ABR; in contrast, municipalities with a higher density of on-premises alcohol experienced a higher ABR.

**Conclusion:**

Our findings highlight the importance of economic drivers and the need to invest in infrastructure, such as pharmacies, medical facilities, schools, and recreation areas and limit the availability of alcohol outlets to increase the impact of current adolescent pregnancy prevention programs.

**Supplementary Information:**

The online version contains supplementary material available at 10.1186/s12889-023-16251-0.

## Introduction

Adolescent pregnancy poses a significant health risk for both mothers and newborns. In addition, it has potentially detrimental effects on the educational and professional opportunities of women [1]. The adolescent birth rate (ABR) is a progress indicator for the United Nations’ Sustainable Development Goals (SDG) targeting health, wellbeing, and gender equity [2]. In 2018, the ABR in México was 70.5 births per 1,000 women aged 15–19, ranging from 48.7 to 96.5 across states, indicating large heterogeneity within the country [3]. This rate decreased from 77 births per 1000 women 15–19 in 2014 [4]. Despite the implementation of successful policies targeting sexual education and contraceptive access, the rate of decline will not be enough to reach the 2030 goal of halving ABR [5, 6], suggesting that other contextual factors could be involved in the causal pathway of adolescent pregnancy.

Using the social determinants framework as an approach to adolescent health, we find two main levels at which social determinants of health operate: the structural and the proximal [7]. Structural determinants influence the environment upon which individual decision making occurs, thus, determining individual choices and behaviors by creating economic, education, political, and social individual and neighborhood stratification systems, which have been strongly associated with overall health outcomes in adolescents [7–11]. The proximal determinants are circumstances of daily life, and include individual-level behaviors and relationships, such as knowledge and use of contraceptives which are targeted by adolescent pregnancy prevention programs [12]. Since structural determinants provide a framework for proximal determinants to occur, it is expected for the improvement of the proximal determinants, such as individual behavior, to require the presence of enabling structures and environments to facilitate this change. Research and policy focus on these enabling structures in adolescent health has been scarce in LMIC [8, 13].

Enabling structures and environments are a critical aspect of adolescent health. Sommer, et al., developed a framework for sexual and reproductive health outcomes, that included the availability and accessibility to resources, such as education and health care, but also neighborhood built environments more broadly [8]. Empirical studies suggests that adolescents living in deteriorating neighborhoods, which are also related to income and gender inequalities, are more vulnerable to sexual risk behaviors [10, 11]. In contrast, living in places with high availability of family planning services physically located within neighborhoods is associated with more contraceptive use among adolescents [14]. Exploring other physical enabling structures could point towards potential interventions to further reduce adolescent pregnancy.

As on other LMIC, fast urbanization rates in Mexico, have resulted in a disorganized growth of cities with large heterogeneities in the built and social environment [15]. Mexico is characterized by important inequities in the distribution of health and education facilities, having a higher concentration in more developed compared to less developed areas [16]. This heterogeneity provides an opportunity to study how characteristics of the physical environment are associated with adolescent sexual and reproductive health outcomes. Understanding these associations would inform future interventions to improve adolescent health through urban planning.

Using a harmonized dataset including city and municipal characteristics from 92 cities in Mexico, we investigated whether the availability, and changes over time in availability, of service facilities or amenities (SFA) is associated with the ABR. We investigated two categories or services/amenities: services/amenities that would support the adoption of healthy adolescent behaviors (schools, primary health-care facilities, pharmacies, and recreation areas such as libraries and museums); and services/amenities that are potentially associated with risk behaviors (off and on-premises alcohol outlets).

## Methods

Data included in this study were retrieved from different sources (explained below) and linked to city or sub-city levels by the *Salud Urbana en America Latina* Project (SALURBAL), which has compiled and harmonized health, social and built-environment data from 371 cities (population ≥ 100,000 in 2010) in eleven countries [17]. Each city is composed by administrative sub-units (ie, *municipios, comunas, distritos, partidos, delegaciones, cantones or corregimientos*). This study includes 406 municipalities from 92 cities that had available information from vital statistics registries from 2008 to 2017 in Mexico. 37 cities are composed of only one municipality.

The outcome of interest was ABR, defined as the total number of live births per 1000 women aged 15 years to 19 years. We assessed ABR at the municipality (ie sub-city) level to capture heterogeneity within larger cities composed of multiple municipalities (*n* = 55 cities). We include yearly estimates from 2008–2017 retrieved from vital statistics registration linked to city and municipality levels based on the mother’s place of residence at the time of the newborn’s birth. Data regarding the population of adolescents 15–19 years old living in each municipality was retrieved using Mexico’s population projections based on census data. After estimating the rates, this variable was categorized in quartiles for the descriptive analysis, with the first quartile corresponding to the lowest rates.

The main exposure of interest was the availability of SFA at the municipal level, which corresponds to the built environment and neighborhood layout in Sommer et al., framework [8]. This information was retrieved from the National Statistical Directory of Economic Units (DENUE) for 2010, 2015 and 2020. DENUE is an inventory of five million non-itinerant economic units related to manufacturing, commerce and services, their main economic activity and location [18]. The information for this directory is based on the National Economic Censuses, which occur every five years (2009, 2014, 2019 being the latest) coordinated by the National Institute of Geography and Statistics. We identified SFA using the North American Industry Classification System (NAICS) codes summarized in Additional file 1. Six different categories of facilities were created: a) education facilities, including all public and private middle- and high-schools; b) health-care facilities, including all public and private primary care clinics (first level of care facilities); c) pharmacies; d) recreation facilities, including public and private libraries, public and private museums, public and private physical activity centers, and bowling centers; e) off-premises alcohol outlets, including wine, liquor, and beer outlets; f) on-premises alcohol outlets, including bars and nightclubs. These categories were selected based on evidence of individual-level characteristics and behaviors that are associated with a higher risk of adolescent pregnancy summarized in Additional file 2.The number of facilities per municipality was obtained for years 2010, 2015, and 2020. We estimated the density per square kilometer (km^2^) by dividing the number of SFA over the municipality total surface in km^2^. Since we retrieved the data for three different timepoints we used linear interpolation with the Stata® command ipolate [19] to obtain yearly estimates.

Analyses were adjusted for several city and sub-city-level covariates found to be associated with ABR in previous work, [20, 21] and variables capturing aspects of economic growth. City-level variables included: population size, population growth, homicide rates, and per capita gross domestic product (GDP). Population indicators were estimated using country-specific population projections, [22] and homicide rates were obtained from vital registration statistics (ICD10 codes: X85-Y09, Y871). GDP for each city was derived from modelled estimates at the subnational level [23]. City-level variables were all considered as time-variant and retrieved for each year. All variables except GDP were available for the period 2009–2017 (GDP data was available for years 2008–2015), we extrapolated the data for the period 2008–2017 using the Stata® command ipolate which uses linear extrapolation [19].

The municipal level covariates are two scores characterizing the social and economic environment, these variables were included to capture socioeconomic changes over time that could confound the associations of ABR with density of services. The two scores are: living conditions (including indicators of sanitation, overcrowding, and adolescent school attendance) and population educational attainment (including indicators of secondary and university educational attainment). These scores were developed by the SALURBAL project using principal component analysis for previous studies, and have been found to be associated with several health outcomes including ABR and infant mortality in cross-sectional studies [21, 24–26]. Higher scores indicate better socioeconomic environment. Municipal-level variables were also considered as time-variant. These scores were created using data for three different timepoints based on Mexico’s census: 2000, 2010, and 2020 we used linear interpolation with the Stata® command ipolate [19] to obtain yearly estimates. We also included population density defined as population per km^2^ in all the urban patches or administrative area inside the geographic boundary, this variable was available for every year from 2008–2017.

## Statistical analysis

We present the yearly distribution of ABR, and the mean and standard deviation for the density of services/amenities and covariates for each timepoint with observed data (at a city or municipal level depending on the variable). We also present their distribution at baseline by municipality’s adolescent birth rate quartile for the year 2010 (the closest to baseline ABR where all observed variables were available).

To assess the association between the different types of facilities and ABR, we fitted negative binomial hybrid models including city and sub-city as random effects [27]. All variables were treated as time-varying. Each variable was included as the overall-unit mean value (${\beta }_{B}{\overline{X} }_{jk})$ and as deviations over time by subtracting the overall-unit mean form each year’s value $({\beta }_{W}({X}_{ijk}-{\overline{X} }_{jk})$ using the following formula:

$$log\left(\#of\,live\,birth{s}_{ijk}\right)={\beta }_{0}+ {\beta }_{W}\left({X}_{ijk}-{\overline{X} }_{jk}\right)+{\beta }_{B}{\overline{X} }_{jk}+{u}_{0jk}+{\mu }_{00k}$$

This allowed us to separate the effect of changes over time in the availability of services/amenities on changes in ABR within municipalities, from the association of between municipality-differences in availability of services/amenities with between-municipality differences in ABR. To avoid collinearity, we fitted separate models for each group of services/amenities in the following sequence: model 1, including only the density of one group of SFA; model 2, also including population density (because population density is related to density of resources and could also impact ABR through other mechanisms, hence could be a confounder); model 3, a fully adjusted model for each group of SFA including city-level population size, population growth, gross domestic product, homicide rates; and municipality-level population density, living conditions score, and educational attainment score. Furthermore, we explored the effect modification of changes in GDP on the effect of changes in the availability of services/amenities on changes in ABR within municipalities, by fitting a fully adjusted model for each type of facility/amenity including an interaction term between the within-municipality change in facilities and change in GDP. All the models were adjusted for a continuous year centered in 2008 to control for other time trends. We considered a level of statistical significance of 0.05, all analysis were done using the software Stata v.16®.

## Results

**Fig. 1:**
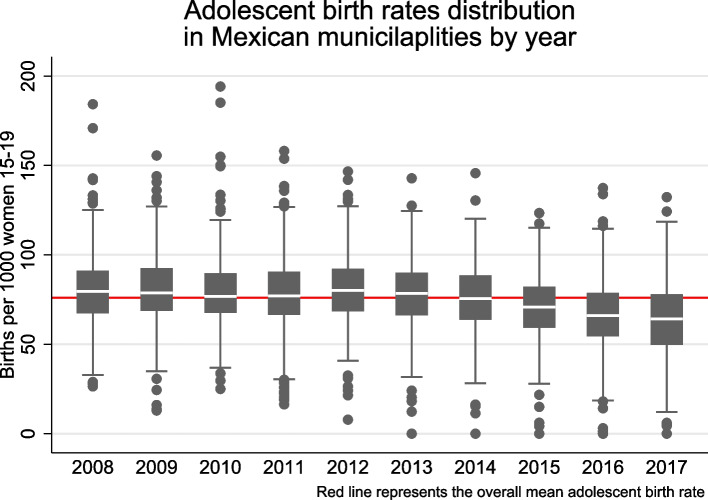
Distribution of adolescent birth rates by year. Each boxplot contains the adolescent birth rates for the 406 municipalities included in the study. The red line corresponds to the mean adolescent birth rate across the study period for all the Mexican cities included

Figure 1 shows the distribution of ABR by year. Overall ABR were stable from 2007–2014 but decreased from 2014–2017. The mean ABR was 80.0 live births per 1,000 women 15–19 years-old in 2008 and decreased to 64.6 in 2017. In addition, we observe large heterogeneity across municipalities with some close or super passing 150 live births per 1,000 women 15–19 years-old, while others remain close to zero live births.

Table 1 presents the distribution of services/amenities and covariates at the different observed timepoints. Overall, the densities of education and recreation facilities and off-premises alcohol outlets statistically significantly increased over time. The density of pharmacies and on-premises alcohol outlets also increased however, this change was not statistically significant. The density of health care facilities remained almost constant over the study period. Between 2010 and 2015 city-level population growth and homicide rates had a statistically significant decrease while GDP had a statistically significant increase. As for the municipality-level socioeconomic scores, both show statistically significant increases over time.

**Table 1:** Distribution of the density of service facilities and amenities per Km^2^ and covariates at different observed timepoints

	**mean (sd)**	**mean (sd)**	**mean (sd)**	***P* value**
**Services and facilities (N per Km**^**2**^**)**	**2010**	**2015**	**2020**	
Education	0.29 (0.60)	0.37 (0.74)	0.41 (0.75)	0.039
Health care	0.51 (1.39)	0.53 (1.36)	0.54 (1.33)	0.955
Pharmacies	0.72 (1.66)	0.90 (1.99)	0.99 (2.11)	0.134
Recreation	0.19 (0.49)	0.33 (0.70)	0.40 (0.78)	< 0.001
Off-premises alcohol outlets	0.34 (0.66)	0.34 (0.63)	0.45 (0.81)	0.045
On-premises alcohol outlets	0.26 (0.26)	0.29 (0.74)	0.34 (0.78)	0.293
**City-level covariates**^a^	**2010**	**2015**		
Population size (100,000)	47.75 (76.34)	49.95 (79.10)		0.989
Population growth (%)	18.17 (7.35)	7.01 (2.52)		< 0.001
Homicide rate (per 100,000 people)	18.42 (25.61)	16.93 (14.55)		< 0.001
Gross domestic product (1000 US. Dollars)	14.56 (8.62)	15.27 (9.01)		0.030
**Municipality level covariates**	**2000**	**2010**	**2020**	
Living conditions score	-3.48 (3.12)	-0.03 (2.21)	1.24 (1.82)	< 0.001
Educational attainment score	-1.34 (1.62)	-0.37 (1.85)	1.13 (2.04)	< 0.001
Population density (1000 per km^2^)^a^		5.02 (2.65)	5.78 (2.76)	< 0.001

^a^These variables are available yearly for the period of 2008–2017, for the purposes of this table we summarized the timepoints that match other observed variables

Table 2 presents the distribution of the density of services/amenities and covariates by adolescent birth rate quartiles in 2010. All the densities of services and facilities presented a similar pattern with higher densities in the municipalities within the lowest quartiles of ABR compared to municipalities within the highest quartiles, with densities being between 2 and 5 times higher in the first quartile compared to the fourth. City-level covariates did not vary systematically across ABR quartiles. Municipality-level living conditions and educational attainment were higher in municipalities within the lowest quartiles compared to the highest quartiles of ABR.

**Table 2:** Distribution the number of service facilities/amenities per km2 and city and municipality level covariates overall and by adolescent birth rate quartiles for year 2010

	**Overall**	**First (lowest)**	**Second**	**Third**	**Forth (highest)**	
	**(25.0–194.1)**	**(25.0–67.6)**	**67.9–76.7)**	**(76.9–89.6)**	**(89.6–194.1)**	***P* value**
N sub-cities	406	102	101	102	101	
N cities^a^	92	34	55	46	41	
**Facilities and services per Km2 (mean, sd)**						
Education	0.29 (0.60)	0.62 (0.94)	0.20 (0.39)	0.18 (0.45)	0.15 (0.22)	< 0.001
Health care	0.51 (1.39)	1.20 (2.41)	0.33 (0.70)	0.31 (0.87)	0.20 (0.29)	< 0.001
Pharmacies	0.72 (1.66)	1.55 (2.50)	0.45 (1.06)	0.49 (1.54)	0.38 (0.66)	< 0.001
Recreation	0.19 (0.49)	0.46 (0.80)	0.12 (0.26)	0.11 (0.37)	0.08 (0.17)	< 0.001
Off-premises alcohol outlets	0.34 (0.66)	0.72 (1.04)	0.28 (0.49)	0.19 (0.35)	0.18 (0.31)	< 0.001
On-premises alcohol outlets	0.26 (0.64)	0.53 (1.04)	0.26 (0.52)	0.15 (0.39)	0.12 (0.20)	< 0.001
**City-level covariates (mean, sd)**						
Population size (100,000)	4.78 (7.63)	6.28 (8.64)	3.15 (6.20)	4.10 (6.94)	5.56 (8.22)	0.015
Population growth (%)	18.4 (25.61)	14.3 (10.04)	18.66 (22.54)	20.98 (37.25)	19.76 (24.89)	0.268
Homicide rate (per 100,000 people)	18.17 (7.35)	17.64 (4.61)	17.82 (7.04)	18.59 (5.45)	18.63 (10.82)	0.685
Gross domestic product (1000 US. Dollars)	14.46 (8.62)	14.32 (6.33)	14.56 (10.92)	14.40 (10.69)	14.52 (5.08)	0.997
**Municipality level covariates (mean, sd)**						
Living conditions score^b^	-0.03 (2.21)	1.32 (2.13)	0.06 (2.02)	-0.53 (2)	-0.96 (2.02)	< 0.001
Educational attainment^c^	-0.37 (1.85)	1.19 (2.19)	-0.27 (1.61)	-1.13 (1.18)	-1.27 (1.09)	< 0.001
Population density (1000 per Km^2^)	5.02 (2.65)	6.20 (3.45)	5.00 (2.22)	4.61 (2.62)	4.35 (1.61)	< 0.001

^a^Some cities have more than 1 municipality (*n* = 55),so the same city may be represented in different columns by different municipalities

^b^Living conditions Score includes % of households with piped water in the dwelling, % of households with overcrowding (3 + per room) in the house, and % of population aged 15–17 attending school

^c^Population- level educational attainment Score includes: % population aged 25 years or above with complete high school level or above, % population aged 25 years or above with complete university level or above

The results of the hybrid models assessing the associations between the density of facilities and amenities and ABR are presented in Table 3. Over the period 2008–2017, increases in densities of pharmacies, recreation centers, and off-premises alcohol outlets were statistically significantly associated with decreases in ABR in unadjusted and adjusted models, with the associations becoming weaker when including covariates (within municipality effects). In adjusted models a 1-unit increase in the density of pharmacies was associated with a 4% decrease in ABR in model 3 (RR 0.96; 95%CI 0.93, 1.00); a 1-unit increase in the density of recreation centers was associated with a 5% decrease in ABR, (RR 0.95; 95%CI 0.90, 0.99) and a 1-unit increase in the density of off-premises alcohol outlets was associated with a 12% decrease in ABR in model 3 (RR 0.88 95%CI 0.82, 0.95). There was an inverse association for the increase in education facilities and a positive one for health care centers and on-premises alcohol outlets with changes in ABR, however, they remained statistically non-significant in all models.

**Table 3:** Rate ratios of ABR associated with within and between municipality differences in densities per km^2^ of services/amenities 2008–2017

	**Within municipality effects (longitudinal)**			**Between municipality effects (cross-sectional)**		
	**Model 1**	**Model 2**^a^	**Model 3**^b^	**Model 1**	**Model 2**^a^	**Model 3**^b^
	**RR (95%CI)**	**RR (95%CI)**	**RR (95%CI)**	**PR (95%CI)**	**PR (95%CI)**	**PR (95%CI)**
Education	0.94 (0.87,1.00)	0.93 (0.87,1.00)	0.92 (0.88,1.01)	0.86 (0.85,0.87)	0.88 (0.86,0.90)	0.97 (0.95,0.98)
Health care	1.04 (0.98,1.10)	1.05 (0.99,1.12)	1.00 (0.95,1.05)	0.95 (0.94,0.95)	0.97 (0.96,0.97)	0.99 (0.98,1.00)
Pharmacies	0.95 (0.92,0.99)	0.95 (0.92,0.98)	0.96 (0.93,1.00)	0.96 (0.95,0.96)	0.98 (0.97,0.99)	0.99 (0.98,1.00)
Recreation	0.93 (0.89,0.98)	0.93 (0.88,0.97)	0.95 (0.90,0.99)	0.88 (0.86,0.90)	0.88 (0.87,0.90)	0.97 (0.95,0.98)
Off-premises alcohol outlets	0.86 (0.78,0.94)	0.86 (0.79,0.94)	0.88 (0.82,0.95)	0.88 (0.87,0.89)	0.93 (0.92,0.95)	1.00 (0.98,1.01)
On-premises alcohol outlets	1.01 (0.96,1.07)	1.02 (0.96, 1.08)	1.03 (0.98,1.09)	0.92 (0.91, 0.93)	0.97 (0.96,0.98)	1.02 (1.01,1.03)

Each row corresponds to a different set of models, modelling each service facility/amenity separately. The effects represent the change in 1 unit per km2 in the density of each service facility/amenities. The standard deviations of the distributions were education 0.60; health care 1.39; pharmacies 1.66; recreation 0.49; and alcohol 0.66

All models were adjusted for year

Gray values reflect non-significant associations

^a^Model 2 only adjusted for municipality population density

^b^Model 3 adjusted for: city-level population size, population growth, gross domestic product, homicide rates; and municipality-level population density, living conditions score, and educational attainment score. All variables were included as time-variant including both the overall mean and the yearly deviation from the mean

Regarding the between municipality effects, all the densities show significant associations in unadjusted and adjusted models, except for off-premises alcohol outlets. We found that a 1-unit higher density of educational and recreational facilities across municipalities was associated with a 3% lower ABR in model 3 (PR 0.97; 95%CI 0.95, 0.98). A 1-unit higher density of health care facilities and pharmacies was associated with 1% lower ABR (PR 0.99; 95% CI 0.98, 1.00) after adjusting for covariates. A 1-unit higher density of on-premises alcohol outlets was associated with 2% higher ABR (PR 1.02 95%CI 1.01, 1.03) The density of off-premises alcohol outlets had an inverse association with ABR in unadjusted models, however, the association weakened and became non-significant after adjustment.

The results of the within-municipality interaction of the density of facilities and GDP are presented in Additional file 3. We found statistically significant interactions for the density of education, health care, pharmacies, and recreational facilities, where increases in GDP over time reduced the effect of increases in the density of facilities on ABR. We did not observe effect modification of GDP changes on off premises and on premises alcohol outlet change within-municipalities.

## Discussion

We investigated if the availability and changes in availability of SFA were associated with ABR in Mexican cities. We found that during the period of 2008–2017 increases in the density per km^2^ of pharmacies, recreation facilities, and off-premises alcohol outlets were associated with decreases in ABR after adjustment for potential confounders. In addition, we found that municipalities with higher densities of education, health care, recreation facilities, and pharmacies had lower ABR while municipalities with a higher density of on-premises alcohol outlets had higher ABR. Our results highlight the importance of availability of SFA as part of interventions to reduce adolescent pregnancy. These results should be carefully interpreted since we did not include any individual-level data in our study and municipal effects might not reflect what happens to everyone within the municipality.

In 2015, Mexico implemented the National Strategy for Adolescent Pregnancy Prevention (ENAPEA, by its Spanish acronym), largely focused on improving comprehensive sexual education and increase contraceptive access though adolescent friendly services [6]. While there has been a decrease in ABR since its implementation, it is happening at a slower rate than needed to achieve the Sustainable Development Goals of reducing ABR by half [3, 28]. Expanding adolescent pregnancy prevention programs beyond education and health care is imperative to increase the rate of decline [5]. Summers et al., [8] framework for adolescent reproductive health behaviors expands from individual and social factors and includes the built and social environment as enablers of healthy and risky adolescent behaviors. Research regarding neighborhood factors associated with adolescent pregnancy has largely focused on social determinants, neighborhood deprivation from an economic standpoint, and segregation, [29, 30] but it has not explored other features of the urban environment. In our study we focused on the structural availability of service facilities and other amenities that could prevent or promote adolescent pregnancy. Our results, even though more research is needed, point towards other options to improve current adolescent pregnancy prevention programs extending beyond individual and social interventions.

The economic situation of LMIC such as Mexico is continuously fluctuating, the turnover of governments and different ideologies in power prioritize resources in different ways at a national level which is also reflected at a local level [31]. During the study period there were significant changes in the density of all service facilities, except medical facilities. Research on contextual factors associated with ABR in other LA countries shows that municipal poverty, violence, and social inequity are associated with higher odds of adolescent pregnancy [32]. Evidence also suggests that living in more deprived neighborhoods is associated with higher risk of adolescent unhealthy behaviors [10, 30]. The overall increase in the densities of pharmacies, recreation facilities, and even off-premises alcohol outlets found in our study reflect important economic changes in municipalities. An increase in the density of pharmacies, recreation areas, and off-premises alcohol outlets, largely driven by chain pharmacies and sport clubs of the private sector, might reflect higher investment and economic growth which could lead to less deprived municipalities. Furthermore, we found that as GDP increased within municipalities, the effect of the increases on the availability of educational, health care, and recreation facilities on ABR become weaker. This suggests that municipalities where GDP was not increasing benefited more from the availability of such services and amenities. While more evidence is needed, our results suggest that allocating resources and investing in infrastructure, particularly in municipalities where economic growth is not increasing, is a potential alternative to prevent adolescent pregnancy over time.

Health care and education have consistently been associated with adolescent pregnancy [33]. They are systematically targeted by adolescent pregnancy prevention programs through the implementation of comprehensive sex education and increasing contraceptive access and counseling [34]. In Mexico, many adolescents get contraception in pharmacies, [35] making them an important extension of the health care system. Yet, health care and education are not the only pathways to reduce ABR. Neighborhoods with museums and libraries tend to have more cultural environments that promote the engagement of young people in such activities [36]. Exposure to art has been found to be therapeutic and help young people deal with past experiences of trauma leading to more healthy behaviors [37]. Also, evidence suggests that physical activity during adolescence might promote resilience and improve self-esteem, found to be associated with a lower risk of adolescent risk behaviors [38, 39]. The involvement of communities in cultural or sports activities promotes social and family cohesion which has shown to be associated with healthier adolescent sexual behaviors [40]. It is difficult to disentangle these effects from the economic characteristics of the municipalities, more economically developed municipalities tend to invest more in education and health care while also receiving more commercial investment from the industry [41]. Even though we did not evaluate the quality of education or health care, nor the services being offered by recreation places, our results suggest that allocating resources to increasing access could potentially reduce ABR.

Evidence from high-income countries suggests that a higher density of alcohol outlets and night clubs is associated with an increased risk of underage drinking and binge-drinking, [42–44] behaviors associated with a higher risk of adolescent pregnancy. We found that municipalities with higher densities of on-premises alcohol outlets had higher ABR. On-premises outlets have been associated with practices such as unsafe sex more commonly by enabling binge drinking and drinking special offers [45]. Also, off-premises outlets tend to prevent selling to minors more than on-premises outlets [46]. We found that an increase in the density of off-premises alcohol outlets was associated with lower ABR. Due to off-premises alcohol outlets data availability, we were not able to include all formal and informal outlets that sell alcoholic beverages, such as some small stores (*tiendas de abarrotes in Mexico*) which have been found to sell alcohol to minors more commonly [47, 48]. These results should be interpreted with caution and further explored by including informal off-premises alcohol outlets that are more likely to sell to minors and better measures to capture the changes in social environment over time.

Our study has some limitations. All the variables obtained from DENUE were interpolated given that we only had 3 time-points, in addition some of our covariates derived from census data were also interpolated. While linear interpolation is a common practice in longitudinal studies and our models were adjusted for year, it is important to keep this in mind when interpreting our results. The densities of services/amenities are highly correlated (Additional file 4) making it hard to disentangle the effects from one another and limiting the inclusion of all of them in the same model. This also suggests that our measures might be a marker of overall investment, and municipality socioeconomic status more than with the specific change on the density of service facilities/amenities; richer municipalities usually allocate more money to the development of education and health care facilities, as well as art and recreation. The data used to estimate adolescent birth rates derives from birth registration, while for the entire country it is more than 95%, [49] it is possible that there are some municipalities with lower registration of births which could bias our results. We used the municipality surface in km^2^ to estimate densities, while most of the municipal boundaries remained constant over our study period, it is possible some boundaries changed which would impact our results. Lasty, our study is subject to the ecologic fallacy given the lack of individual-level data which warrants careful interpretation of our results as municipal effects might not reflect what happens to everyone within the municipality. Additionally, given the use of aggregated data, we did not account for individuals who might reside in one municipality but access/utilize services in another.

Using the social determinants of health and the structural behavior model, we analyzed the structural sphere at a municipal level which is often under looked. Our findings highlight the association of economic drivers and adolescent birth rates. Furthermore, they point towards the need to invest in infrastructure, such as pharmacies, medical facilities, schools, and recreation areas and limit the availability of alcohol outlets to potentially increase the impact of current adolescent pregnancy prevention programs. While we should consider that our findings might not be directly associated with a decrease in adolescent pregnancy but could indirectly affect the behavioral pathways associated with it, further research is needed to identify more factors within city infrastructure that are associated with youth and adolescent health outcomes. These results suggest that local governments could expand national programs and increase their reach by potentially investing in structural expansion that would benefit not only adolescents but the entire community.

## Supplementary Information

**Additional file 1: Table S1. **North American Industry Classification System (NAICS) selected from DENUE to create exposure variables.

**Additional file 2: Table S2.** Characteristics and behaviors associated with higher risk of adolescent pregnancy.

**Additional file 3: Table S3.** Rate ratios of ABR associated with within municipality main effects and interaction of densities per km^2^ of services/amenities and city-level GDP 2008-2017.

**Additional file 4: Table S4.** Correlation of the density of service facilities and amenities per km^2^.

## Data Availability

The datasets generated and/or analyzed during the current study are available in the National Institute of Geography and Statistics of Mexico (INEGI) repository, Live births: https://www.inegi.org.mx/programas/natalidad/ Population projections: https://www.inegi.org.mx/temas/estructura/ Data from the Economic Census: https://en.www.inegi.org.mx/app/mapa/denue/ Census data: https://www.inegi.org.mx/programas/ccpv/2020/

## Abbreviations

ABR: Adolescent Birth Rates
SDG: Sustainable Development Goals
GDP: Gross Domestic Product per capita
LMIC: Lowand Middle-Income Countries
DENUE: National Statistical Directory of Economic Units
RR: Rate Ratio
95% CI: 95% Confidence Interval
SALURBAL: Salud Urbana in América Latina
