# Supplementary material for: Association of service facilities and amenities with adolescent birth rates in Mexican cities
Source: BMC Public Health. 2023 Jul 10;23:1321. doi: 10.1186/s12889-023-16251-0 (PMC10334546; doi:10.1186/s12889-023-16251-0)
Supplement: Supplementary file 1 — Additional file 1: Table S1. North American Industry Classification System (NAICS) selected from DENUE to create exposure variables. [file 12889_2023_16251_MOESM1_ESM.docx]

**Table S1.** North American Industry Classification System (NAICS) selected from DENUE to create exposure variables

| **Service facility/amenity** | **NAICS code** | **Description** |
| --- | --- | --- |
| Pharmacies | 464111 | Pharmacies with supermarket |
|  | 464112 | Pharmacies without supermarket |
| Education | 611131 | Private sector general secondary education schools |
|  | 611132 | Public sector general secondary education schools |
|  | 611141 | Private sector technical secondary education schools |
|  | 611142 | Public sector technical secondary education schools |
|  | 611151 | Private sector technical middle school |
|  | 611152 | Public sector technical middle school |
|  | 611161 | Private sector high school education schools |
|  | 611162 | Public sector high school education schools |
|  | 611171 | Private sector schools combining different levels of education (primary, secondary, high school) |
|  | 611172 | Public sector schools combining different levels of education (primary, secondary, high school) |
| Health care | 621111 | Private sector general medicine consultation office |
|  | 621112 | Public sector general medicine consultation office |
|  | 621115 | Private sector general medicine clinics |
|  | 621116 | Public sector general medicine clinics |
|  | 621411 | Private sector family planning centers |
|  | 621412 | Public sector family planning centers |
| Recreation | 519121 | Private sector libraries |
|  | 519122 | Public sector libraries |
|  | 712111 | Private sector museums |
|  | 712112 | Public sector museums |
|  | 713941 | Private sector sports clubs |
|  | 713942 | Public sector sports clubs |
|  | 713943 | Private sector physical conditioning centers |
|  | 713944 | Public sector physical conditioning centers |
|  | 713950 | Bowling centers |
| Off-premises alcohol outlets | 461211 | Wine and liqueur outlets |
|  | 461212 | Beer outlets |
| On-premises alcohol outlets | 722411 | Centros nocturnos, discotecas |
|  | 722412 | Bares, cantinas |
