# Supplementary material for: Association of service facilities and amenities with adolescent birth rates in Mexican cities
Source: BMC Public Health. 2023 Jul 10;23:1321. doi: 10.1186/s12889-023-16251-0 (PMC10334546; doi:10.1186/s12889-023-16251-0)
Supplement: Supplementary file 2 — Additional file 2: Table S2. Characteristics and behaviors associated with higher risk of adolescent pregnancy. [file 12889_2023_16251_MOESM2_ESM.docx]

**Table S2.- Characteristics and behaviors associated with higher risk of adolescent pregnancy**

| **Category of service facility/amenity** | **Individual-level behavior/factor** | **Association with adolescent pregnancy/birth rates** |
| --- | --- | --- |
| Education facilities | Provide education including sexual and reproductive health. | Higher education is associated with lower rates of adolescent birth rates. Also associated with delaying pregnancy.(1,2) |
| Health care | Provide sexual reproductive health care and contraceptives | Higher access to reproductive health services and contraception is associated with lower adolescent birth rates.(1) |
| Pharmacies | Provide contraceptive access (in Mexico no prescription or parental consent is required) | Higher access to contraception is associated with lower adolescent birth rates. (3) |
| Recreation facilities | Provide safe spaces to release stress, anxiety and create healthy behaviors. | Physical activity, and exposure to art are associated with healthier lifestyles and lower rates of adolescent risk behaviors.(4,5) |
| On/off premises alcohol outlets | Access and advertisement to alcohol | Associated with binge drinking and other adolescent risk behaviors which increase the risk of adolescent pregnancy. (6–8) |

References:

1. Caffe S, Plesons M, Camacho A V, Brumana L, Abdool SN, Huaynoca S, et al. Looking back and moving forward: can we accelerate progress on adolescent pregnancy in the Americas? Reprod Heal [Internet]. 2017;14(1):83. Available from: https://www.ncbi.nlm.nih.gov/pubmed/28705166

2. Rodríguez Ribas C. Adolescent pregnancy, public policies, and targeted programs in Latin America and the Caribbean: a systematic review. Rev Panam Salud Publica;45, dec 2021 [Internet]. 2021 Dec 9 [cited 2022 Jan 11];45:1. Available from: https://iris.paho.org/handle/10665.2/55342

3. Gómez-Inclán S, Durán-Arenas L. El acceso a métodos anticonceptivos en adolescentes de la Ciudad de México. Salud Publica Mex [Internet]. 2017 May 1 [cited 2022 Jan 27];59(3):236–47. Available from: http://doi.org/10.21149/7891

4. Hartz L, Thick L. Art Therapy Strategies to Raise Self-Esteem in Female Juvenile Offenders: A Comparison of Art Psychotherapy and Art as Therapy Approaches. http://dx.doi.org/101080/07421656200510129440 [Internet]. 2011 Jan 1 [cited 2022 Jan 27];22(2):70–80. Available from: https://www.tandfonline.com/doi/abs/10.1080/07421656.2005.10129440

5. Belcher BR, Zink J, Azad A, Campbell CE, Chakravartti SP, Herting MM. The Roles of Physical Activity, Exercise, and Fitness in Promoting Resilience During Adolescence: Effects on Mental Well-Being and Brain Development. 2021 [cited 2022 Jan 27]; Available from: https://doi.org/10.1016/j.bpsc.2020.08.005

6. Puac-Polanco V, Keyes KM, Mauro PM, Branas CC. A Systematic Review of Drink Specials, Drink Special Laws, and Alcohol-Related Outcomes. Curr Epidemiol Reports 2020 74 [Internet]. 2020 Oct 31 [cited 2022 Feb 9];7(4):300–14. Available from: https://link.springer.com/article/10.1007/s40471-020-00247-0

7. Fairman BJ, Goldstein RB, Simons-Morton BG, Haynie DL, Liu D, Hingson RW, et al. Neighbourhood context and binge drinking from adolescence into early adulthood in a US national cohort. Int J Epidemiol [Internet]. 2020 Feb 1 [cited 2022 Feb 9];49(1):103–12. Available from: https://academic.oup.com/ije/article/49/1/103/5526902

8. Chinman M, Burkhart Q, Ebener P, Fan CC, Imm P, Osilla KC, et al. The Premises is the Premise: Understanding Off- and On-premises Alcohol Sales Outlets to Improve Environmental Alcohol Prevention Strategies. Prev Sci [Internet]. 2011 Jun 4 [cited 2022 Feb 9];12(2):181–91. Available from: https://link.springer.com/article/10.1007/s11121-011-0203-z
