# Supplementary material for: Association of service facilities and amenities with adolescent birth rates in Mexican cities
Source: BMC Public Health. 2023 Jul 10;23:1321. doi: 10.1186/s12889-023-16251-0 (PMC10334546; doi:10.1186/s12889-023-16251-0)
Supplement: Supplementary file 3 — Additional file 3: Table S3. Rate ratios of ABR associated with within municipality main effects and interaction of densities per km2 of services/amenities and city-level GDP 2008-2017. [file 12889_2023_16251_MOESM3_ESM.docx]

**Table S3.** Rate ratios of ABR associated with within municipality main effects and interaction of densities per km^2^ of services/amenities and city-level GDP 2008-2017.

|  | **Main effect density** | **Main effect GDP** | **Interaction term** |
| --- | --- | --- | --- |
| **Facilities/Km^2^** | **RR (95%CI)** | **RR (95%CI)** | **RR (95%CI)** |
| Education | **1.31 (1.12,1.52)** | 1.00 (0.96,1.04) | **0.85 (0.80,0.91)** |
| Health care | **1.12 (1.01,1.24)** | 1.00 (0.94,1.06) | **0.94 (0.89,0.99)** |
| Pharmacies | 1.08 (0.99,1.18) | 1.00 (0.94,1.06) | **0.95 (0.92,0.99)** |
| Recreation | **1.16 (1.04,1.30)** | 1.00 (0.94,1.06) | **0.90 (0.85,0.95)** |
| Off premises alcohol outlets | 0.92 (0.80,1.05) | 0.99 (0.94,1.05) | 0.98 (0.91,1.05) |
| On premises alcohol outlets | **1.14 (1.01,1.28)** | 1.00 (0.94,1.06) | 0.94 (0.88,1.00) |

Each row corresponds to a different set of models, modelling each service facility/amenity separately. The effects represent the change in 1 unit per km2 in the density of each service facility/amenities. The standard deviations of the distributions were education 0.60; health care 1.39; pharmacies 1.66; recreation 0.49; and alcohol 0.66.

All models were adjusted for year.

Bolded values reflect statistically significant associations (p < 0.05)

Models adjusted for: city-level population size, population growth, homicide rates; and municipality-level population density, living conditions score, and educational attainment score. All variables were included as time-variant including both the overall mean and the yearly deviation from the mean.
