# Supplementary material for: Association of service facilities and amenities with adolescent birth rates in Mexican cities
Source: BMC Public Health. 2023 Jul 10;23:1321. doi: 10.1186/s12889-023-16251-0 (PMC10334546; doi:10.1186/s12889-023-16251-0)
Supplement: Supplementary file 4 — Additional file 4: Table S4. Correlation of the density of service facilities and amenities per km2. [file 12889_2023_16251_MOESM4_ESM.docx]

**Table S4.** Correlation of the density of service facilities and amenities per km^2^

|  | **Education** | **Health care** | **Pharmacies** | **Recreation** | **Off-premises**  **alcohol outlets** | **On-premises**  **alcohol outlets** |
| --- | --- | --- | --- | --- | --- | --- |
| **Education** | 1 |  |  |  |  |  |
| **Health care** | 0.8931 | 1 |  |  |  |  |
| **Pharmacies** | 0.9466 | 0.8459 | 1 |  |  |  |
| **Recreation** | 0.9457 | 0.8995 | 0.9274 | 1 |  |  |
| **Off-premises alcohol outlets** | 0.6471 | 0.6149 | 0.5884 | 0.6979 | 1 |  |
| **On-premises alcohol outlets** | 0.6692 | 0.766 | 0.5802 | 0.7239 | 0.6872 | 1 |
